# Aromatic Herbs, Medicinal Plant-Derived Essential Oils, and Phytochemical Extracts as Potential Therapies for Coronaviruses: Future Perspectives

**DOI:** 10.3390/plants9060800

**Published:** 2020-06-26

**Authors:** Mohamed Nadjib Boukhatem, William N. Setzer

**Affiliations:** 1 Département de Biologie et Physiologie Cellulaire, Faculté des Sciences de la Nature et de la Vie, Université - Saad Dahlab - Blida 1, BP 270, Blida 09000, Algeria; 2 Department of Chemistry, University of Alabama in Huntsville, Huntsville, AL 35899, USA; wsetzer@chemistry.uah.edu; 3 Aromatic Plant Research Center, 230 N 1200 E, Suite 100, Lehi, UT 84043, USA

**Keywords:** 2019-nCoV, SARS-CoV, MERS-CoV, COVID-19, Severe Acute Respiratory Syndrome Coronavirus 2, herbal medicines, essential oils, phytochemicals, medicinal plants, antiviral activity

## Abstract

After its recent discovery in patients with serious pneumonia in Wuhan (China), the 2019 novel coronavirus (2019-nCoV), named also Severe Acute Respiratory Syndrome Coronavirus 2 (SARS-CoV-2), has spread quickly. Unfortunately, no drug or vaccine for treating human this coronavirus infection is available yet. Numerous options for controlling or preventing emerging 2019-nCoV infections may be predicted, including vaccines, interferon therapies, and small-molecule drugs. However, new interventions are likely to require months to years to develop. In addition, most of the existing antiviral treatments frequently lead to the development of viral resistance combined with the problem of side effects, viral re-emergence, and viral dormancy. The pharmaceutical industry is progressively targeting phytochemical extracts, medicinal plants, and aromatic herbs with the aim of identifying lead compounds, focusing principally on appropriate alternative antiviral drugs. Spices, herbal medicines, essential oils (EOs), and distilled natural products provide a rich source of compounds for the discovery and production of novel antiviral drugs. The determination of the antiviral mechanisms of these natural products has revealed how they interfere with the viral life cycle, i.e., during viral entry, replication, assembly, or discharge, as well as virus-specific host targets. Presently, there are no appropriate or approved drugs against CoVs, but some potential natural treatments and cures have been proposed. Given the perseverance of the 2019-nCoV outbreak, this review paper will illustrate several of the potent antiviral chemical constituents extracted from medicinal and aromatic plants, natural products, and herbal medicines with recognized in vitro and in vivo effects, along with their structure–effect relationships. As this review shows, numerous potentially valuable aromatic herbs and phytochemicals are awaiting assessment and exploitation for therapeutic use against genetically and functionally different virus families, including coronaviruses.

## 1. Introduction

Viruses are responsible for several infections and diseases comprising cancer, while complex disorders such as Alzheimer’s illness and type 1 diabetes have also been linked to virus-related infections [1]. In addition, due to increased foreign travel and rapid urbanization, infectious outbreaks caused by emerging and re-emerging pathogens, including viruses, pose a serious danger to community health care, principally if antiviral treatment and protective vaccines are not available. Up to the present time, several viruses persist without potent immunization, and only limited virucidal molecules are approved for clinical use in humans [2].

In 1937, coronaviruses were identified from poultry and were considered extremely important pathogenic viruses in livestock, causing periodic cold or mild human digestive infections [3]. A new human coronavirus (CoV) became notably popular in spring 2003 because of an outbreak in South-East Asia and Canada [4].

At the time, the suspect virus was quickly recognized as the Severe Acute Respiratory Syndrome-Coronavirus (SARS-CoV) but did not bear a resemblance to the human CoVs. SARS-CoV worried the world because it sickened more than 7500 persons and killed more than 700 of them [5]. It was not until the SARS epidemic of 2002–2003 that research and investigation for particular anti-coronavirus vaccines or therapies started [6].

A novel coronavirus has caused severe mortality associated with a respiratory contagious disease. The virus is named Middle East Respiratory Syndrome Coronavirus (MERS-CoV). This novel MERS-CoV was first observed in different countries including Saudi Arabia [7]. A novel coronavirus with human-to-human contagion and causing a particularly serious illness, occurring in Wuhan, China, was confirmed towards the end of December 2019 [8]. The virus was named SARS-CoV-2 and the disease it causes was named Coronavirus Disease 2019 (abbreviated “COVID-19”).

**Figure 1:**
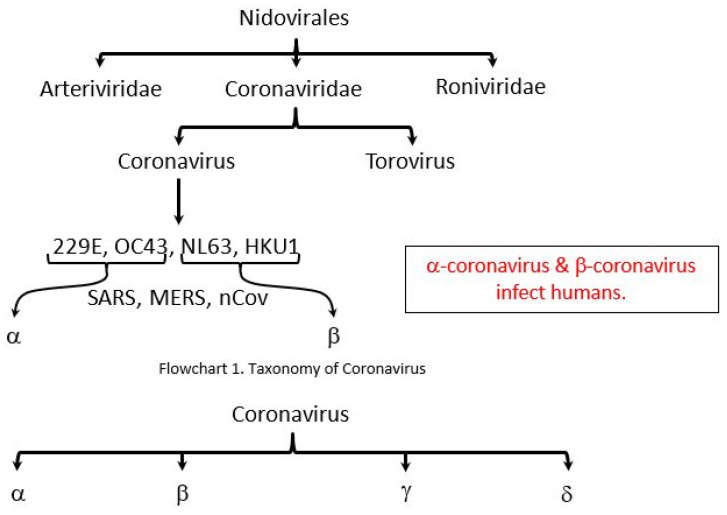
The taxonomy of the order Nidovirales. https://epomedicine.com/medical-students/coronavirus-disease-covid-2019/; (CSSE; FT research; Updated: 17 March 2020, 10:00 GMT). SARS, Severe Acute Respiratory Syndrome, MERS, Middle East Respiratory Syndrome Coronavirus, nCov, novel coronavirus. α: Alpha; β: Beta; γ: Gamma; δ: Delta.

Early on, several of the patients at the epidemic center in Wuhan, Hubei Province (China), had some connection with a vast market of seafood and animals, implying the animal-to-person transmission. Afterward, an increasing number of patients apparently had no access to animal marketplaces, implying transmission from person to person [9]. The coronavirus group comprises numerous species (Figure 1) and induces respiratory tract and gastrointestinal infections in vertebrates; however, some CoVs such as SARS, MERS, and SARS-CoV-2 have been shown to be especially dangerous to humans [10]. Coronaviruses comprise an extensive collection of viruses, which commonly infect humans as well as numerous other mammalian species such as cattle, farm animals, household pets, and bats [11]. Infrequently, coronaviruses could infect humans from animals and subsequently expand among persons, as was observed for MERS-CoV, SARS-CoV, and now with SARS-CoV-2 [12].

There are no effective or approved therapies for CoV diseases, and protective vaccines are still being investigated. Therefore, it is necessary to discover potent antivirals for protection from and management of CoV infection in humans [13]. The novelty of the 2019 novel coronavirus (2019-nCoV) means that there are numerous uncertainties surrounding its behavior; consequently, it is too early to conclude whether herbal and medicinal plants, spices, or isolated compounds and molecules could be used as prophylactic/preventive drugs or as appropriate therapeutic compounds against COVID-19. Nevertheless, due to the high similarity of SARS-CoV-2 with the previously reported MERS-CoV and SARS-CoV viruses, previous research articles on phytomedicine and herbal compounds, which have been demonstrated to have anti-coronavirus properties, may be an appreciated guide to searching and discovering antiviral phytochemical extracts which may be effective against SARS-CoV-2 virus [14,15,16,17,18,19,20,21,22,23,24,25,26,27,28,29,30,31,32,33,34].

Published patent applications and academic investigations on the most relevant compounds and methods for the treatment of coronaviruses are reviewed, focusing on those strategies that attack one particular phase of the development cycle of coronaviruses, because they have greater potential as lead structural templates for further development.

In this review article, we summarize the antiviral properties from numerous phytochemical extracts, aromatic herbs, and medicinal plants against different CoV. These medicinal plants and phytochemical extracts offer an important source for innovative and effective antiviral drug discovery, allowing inexpensive and relatively safe drug development.

## 2. COVID-19 Is Now Officially a Pandemic

The coronavirus 2019-nCoV has infected numerous people in China and spread to other regions in a short period. On 30 January, 2020, the World Health Organization (WHO) confirmed that the epidemic of 2019-nCoV is a global health crisis and delivered initial suggestions [34,35]. On 2 February 2020, and according to China’s National Health Commission’s report, 14,488 clinical infections were found in China, comprising 304 deaths.

As we write this and according to the WHO, COVID-19 threatens 200 nations and regions across the planet and two multinational transports: the luxury ship Diamond Princess harbored in Yokohama, Japan, and the cruise ship MS Zaandam from Holland America [36]. The COVID-19 viral infection resulted in the deaths of more than 182,000 individuals and is now officially considered to be a pandemic. This viral infection is considered to be the first pandemic due to a coronavirus. In addition, it is the first time the WHO has called an infectious outbreak a pandemic since the H1N1 “swine flu” in 2009. Furthermore, different American, Asian, and European countries are now each recording more than 800,000 cases of COVID-19, caused by the 2019-nCoV that has infected more than 5,000,000 people worldwide. In the past three weeks, the number of affected countries has tripled, and the number of human cases of COVID-19 outside China has increased 15-fold. The WHO is profoundly worried, both with the disturbing degrees of seriousness of the infection and the dissemination of the disease and with the disturbing degrees of indecision and complacency of many world leaders in reaction to the epidemic. Therefore, COVID-19 is now recognized as a pandemic. In the previous pandemic, according to the WHO, the H1N1 influenza virus infected more than 18,000 people in more than 214 territories and nations.

## 3. An Overview of COVID-19

The entire medical picture of COVID-19 is not completely known. Recorded illnesses have oscillated from very minor (even those with no clinical symptoms) to serious, including deadly infection. Although clinical reports have shown that most infections with COVID-19 are mild to date, a recent investigation [37] from China indicates that severe illness occurs in 16% of cases. Older individuals and different age groups with serious chronic medical conditions such as respiratory disease, cardiovascular disease, and diabetes tend to be at higher risk of contracting extreme COVID-19 [38,39,40]. As individuals, practicing prevention measures and good hygiene as well as applying actions of social distancing, including avoiding crowded places, remain to be very essential [34,41].

The pandemic is persisting, and discovering innovative prevention and medicinal medicines or vaccinations as early as possible is vital and necessary. In addition, effective measures for early identification, exclusion, and diagnosis of individual patients, as well as reducing exposure and dissemination by social contact and activities must be implemented.

Although successful vaccinations and antiviral medicines are the most potent means of combating or avoiding virus diseases and contaminations, there are no cures yet for 2019-nCoV infection. The creation and production of such medications may take several months or years, thereby indicating the need for finding alternative rapid treatment or control strategies.

## 4. Antiviral Activity of Herbal Medicines and Phytochemicals against Coronaviruses

To this end, aromatic herbs, herbal teas, culinary spices, and medicinal plants used in ethnobotanical treatments may represent highly useful sources. During the 2003 SARS outbreak [16], the efficacy and performance of herbal therapy and phytomedicine for preventing viral infections was illustrated.

As such, different countries, including Algeria, are encouraging the use of herbal and medicinal plants in fighting SARS-CoV-2 infection [15,16,17,21,22,24,25,26,29,30].

After the outbreak of SARS-CoV, first described in early 2003, researchers and scientists have been dynamically trying to explore different antiviral extracts, drugs, and molecules against SARS-CoV. This had led a group of experts to screen more than 200 medicinal plants, culinary spices, and aromatic herbs for their antiviral properties against this SARS-CoV strain [42]. In fact, after the outbreak of SARS, many groups started to search for anti-coronavirus agents, including some natural compounds and phytochemical extracts that exist in traditional herbal medicines [18,21,23,31]. Table 1 presents several studies reporting the inhibitory effect of medicinal plants or isolated compounds on different strains of human coronavirus.

**Figure 2:**
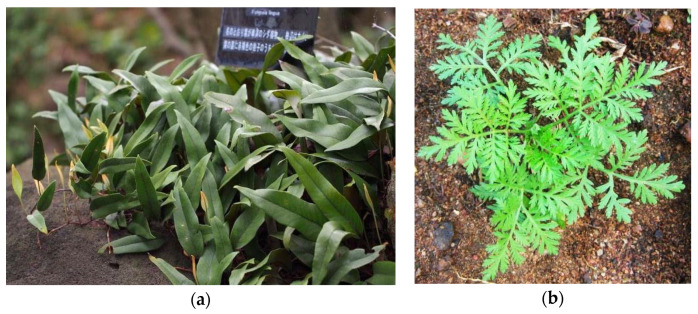
Aromatic plants tested against SARS-CoV: (**a**) *Pyrrosia lingua* [60]; (**b**) *Artemisia annua* [61].

Among these, four extracts exhibited moderate to potent inhibition effects against SARS-CoV: *Lycoris radiata* (red spider lily), *Pyrrosia lingua* (a fern) (Figure 2a), *Artemisia annua* (sweet wormwood) (Figure 2b), and *Lindera aggregata*, which is an aromatic evergreen shrub, member of the laurel family. The antiviral effects of these extracts were dose-dependent and ranged from low to high concentrations of the extracts, depending in the herbal extract considered. In particular, *L. radiata* exhibited the most potent antiviral activity against the virus strain [23].

**Figure 3:**
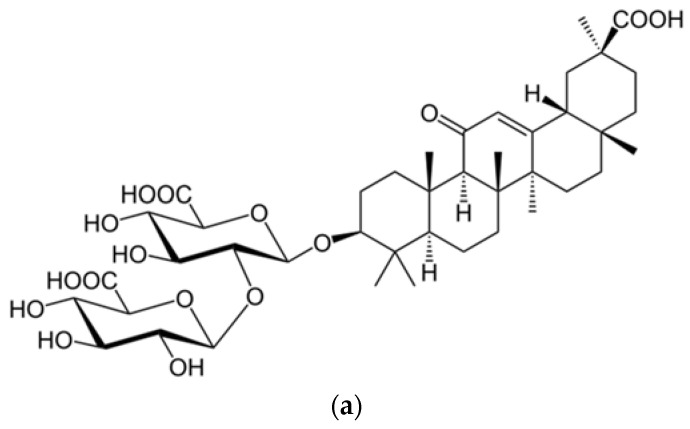

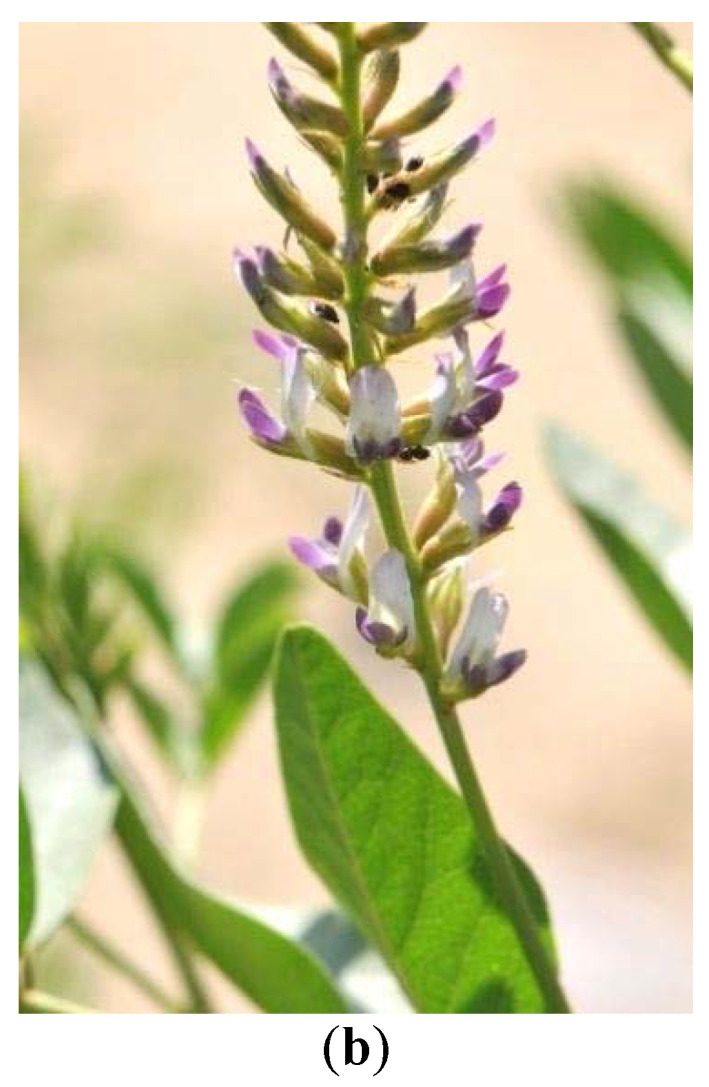
(**a**) Structure of glycyrrhizic acid (glycyrrhizin; glycyrrhizinic acid) [63]; (**b**) *Glycyrrhiza glabra*. [64].

These data are in accordance with those of two other research teams, which confirmed that an active compound contained in licorice roots, i.e., glycyrrhizin (Figure 3a), exerts an anti-SARS-CoV effect by stopping viral replication [18,62]. In another investigation, glycyrrhizin (*Glycyrrhiza glabra*, Fabaceae family) (Figure 2b) also displayed antiviral property when tested for its in vitro antiviral activity on 10 different clinical strains of SARS-CoV.

**Figure 4:**
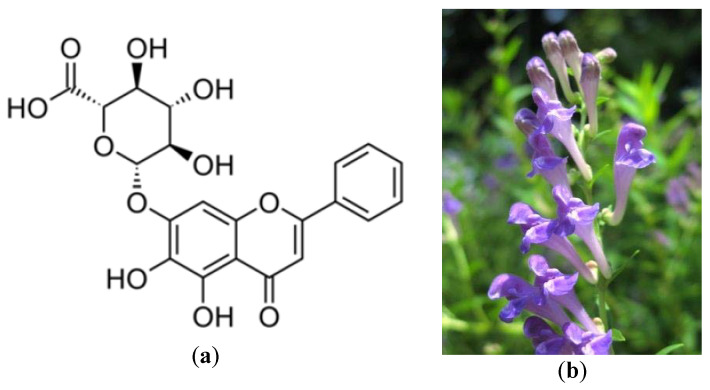
(**a**) Baicalin structural formula: baicalin is a flavone (flavonoid) found in several species of the genus *Scutellaria*, including (**b**) *Scutellaria baicalensis* root [66].

Baicalin (Figure 4a), a constituent of the plant Baikal skullcap (*Scuttelaria baicalensis*) (Figure 4b), was been examined in this research under the same conditions and also revealed antiviral potential against SARS-CoV [15]. Baicalin has also been shown to inhibit the replication of the HIV-1 virus in vitro in previous publications [24,65]. Nevertheless, it should be noted that in vitro findings may not correlate with in vivo clinical efficacy. This is because the oral quantity of these molecules in humans may not attain a blood serum dose comparable to that tested in vitro dose.

**Figure 5:**
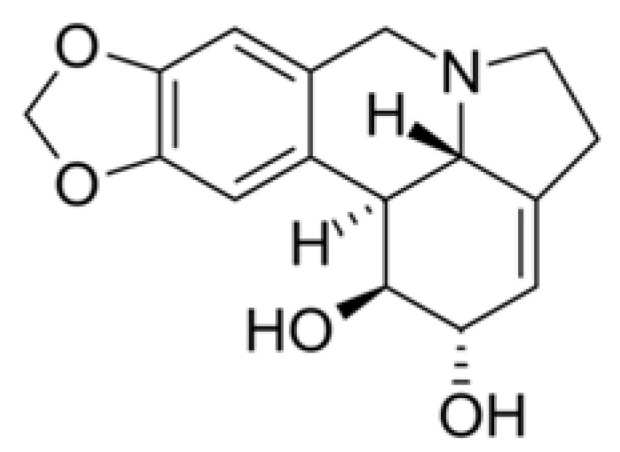
Lycorine chemical structure. It is a toxic alkaloid found in various Amaryllidaceae species (other names: galanthidine, amaryllis, narcissine) [69].

Lycorine (Figure 5) is a toxic crystalline alkaloid found in various Amaryllidaceae species, such as the cultivated bush lily (*Clivia miniata*), surprise lilies (*Lycoris*), and daffodils (*Narcissus*). It has also demonstrated a potent antiviral effect against SARS-CoV. Several previous investigations suggest that lycorine seems to have broad antiviral properties and has been reported to have an inhibitory action on the Herpes simplex virus (HSV, type I) [67] and Poliomyelitis virus [68].

**Figure 6:**
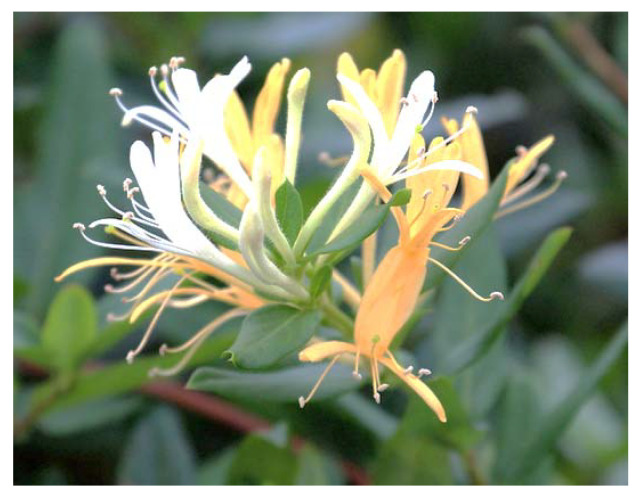
Flowers of honeysuckle (*Lonicera *japonica** Thunb) [70].

**Figure 7:**
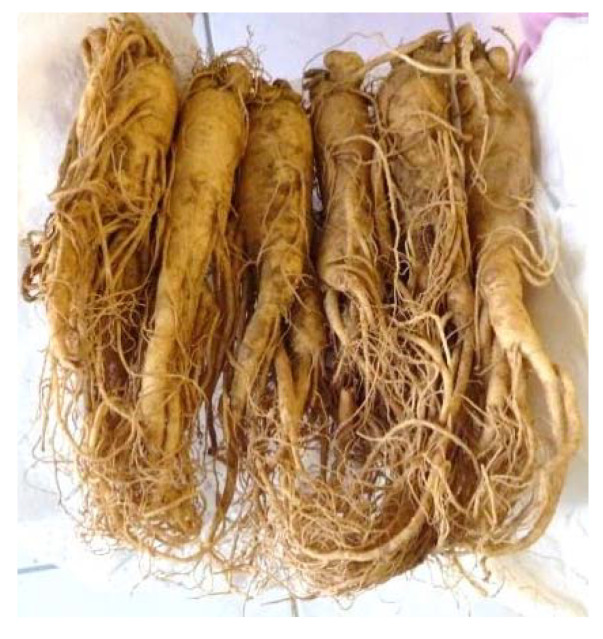
Korean ginseng (*Panax ginseng*) [71].

Other medicinal herbs and plants and culinary spices that have been described to have antiviral properties against SARS-CoV are Japanese honeysuckle (*Lonicera japonica* Thunb.) (Figure 6), the commonly known *Eucalyptus* tree, and Korean ginseng (*Panax ginseng*) (Figure 7), the last one through its active secondary metabolite ginsenoside-Rb1 [31].

**Figure 8:**
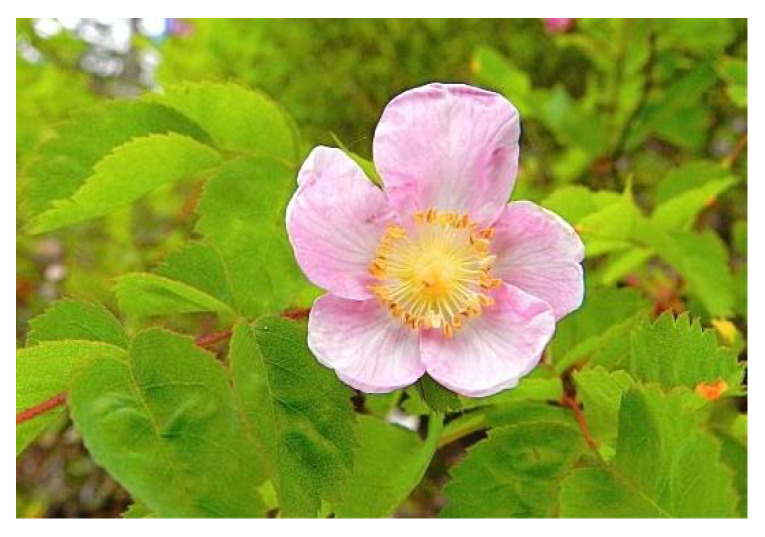
Nookta Rose (*Rosa nutkana*) [72].

**Figure 9:**
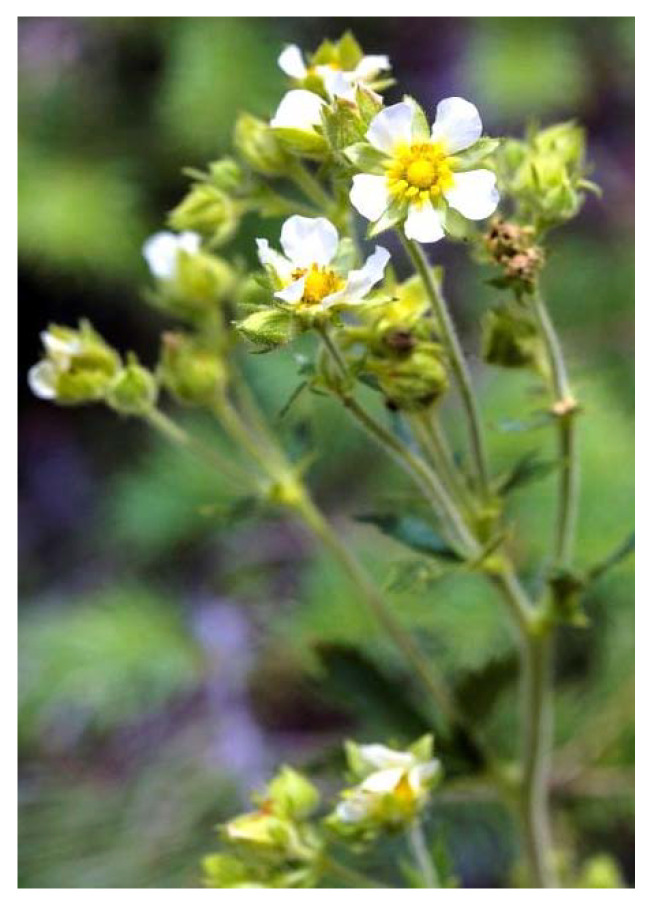
Potentilla arguta [73].

**Figure 10:**
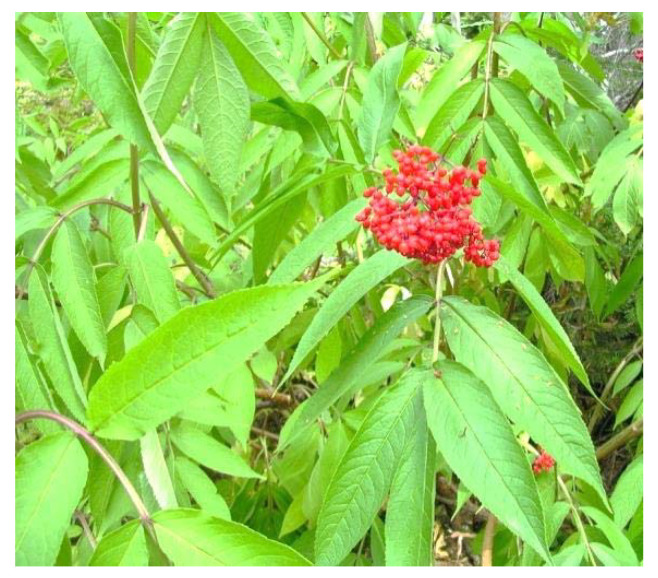
*Sambucus racemosa* (red elderberry) [74].

One hundred British Columbian aromatic and medicinal herbs were evaluated for antiviral effect against seven viruses [29]. Twelve phytochemical extracts were shown to possess antiviral properties at the doses used. The phytochemical extracts of Saskatoon or Pacific serviceberry (*Amelanchier alnifolia*) and Nootka or wild rose (*Rosa nutkana*) (Figure 8) were the most effective against an enteric coronavirus. Respiratory syncytial virus (RSV) was totally blocked by a root extract of tall cinquefoil (*Potentilla arguta*) (Figure 9) and a branch tip extract of red elderberry (*Sambucus racemosa*) (Figure 10).

**Figure 11:**
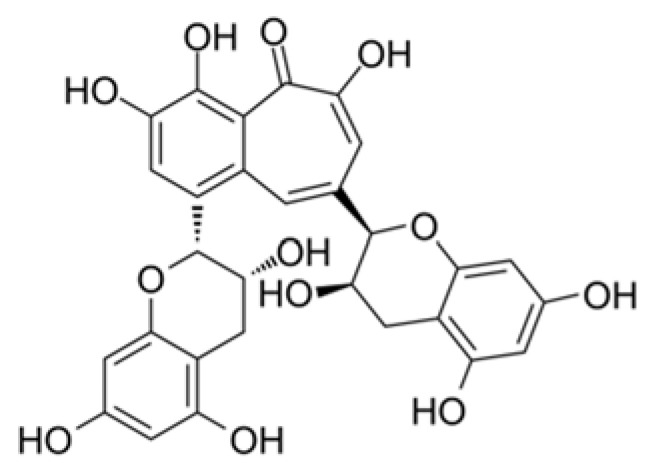
Theaflavin chemical structure. Theaflavin is an effective inhibitor of influenza A (H1N1) neuraminidase [77].

Bioflavonoids derived from herbal medicines have been tested for antiviral properties [75]. The black tea flavonoid theaflavin (Figure 11) has been a well-known antioxidant with free radical-scavenging ability and has been able to neutralize infections of bovine coronavirus [76].

## 5. Mode of Antiviral Action

Many investigations and studies of plant extracts and pure molecules have been carried out with different strains of coronavirus. Proteins involved in coronaviral replication and the conductance of ion channels and proteases were the main targets [78]. Several researchers have discovered plant formulations that inhibit in vivo and in vitro viral replication [79,80].

Evidence from the above-mentioned publications and reports and numerous other international investigations reveal that many phytochemical extracts and medicinal plant constituents have displayed antiviral properties against coronaviruses [29], and their principal mode of action seems to be through the inhibition of viral replication [19]. Otherwise, molecules that have an antiviral activity work like a disinfectant or antiseptic and do not necessitate repetition to inactivate a virus [81]. Resistance to antiviral compounds is probably caused by mutations created in the viral genome in the course of replication [82].

**Figure 12:**
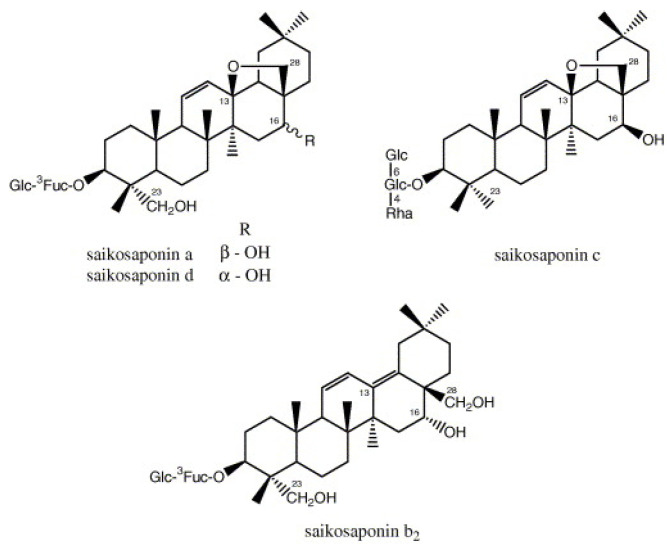
Chemical structures of saikosaponins a, c, and d [25].

It has been experimentally verified that saikosaponins (a, b_2_, c, and d) (Figure 12), which are naturally produced triterpene glycosides isolated from herbal medicines such as Chinese thoroughwax (*Bupleurum* spp., belonging to the family Apiaceae), parsley tree (*Heteromorpha* spp., belonging to the family Apiaceae), and Figwort (*Scrophularia scorodonia*, belonging to the family Scrophulariaceae), have antiviral activity against HCoV-22E9, a species of CoV that infects humans and animals and together with human coronavirus OC43 is one of the common-cold viruses [17]. Such natural molecules effectively suppress and deter the early process of infection by HCoV-229E, including viral penetration and attachment, after co-challenge with the virus.

**Figure 13:**
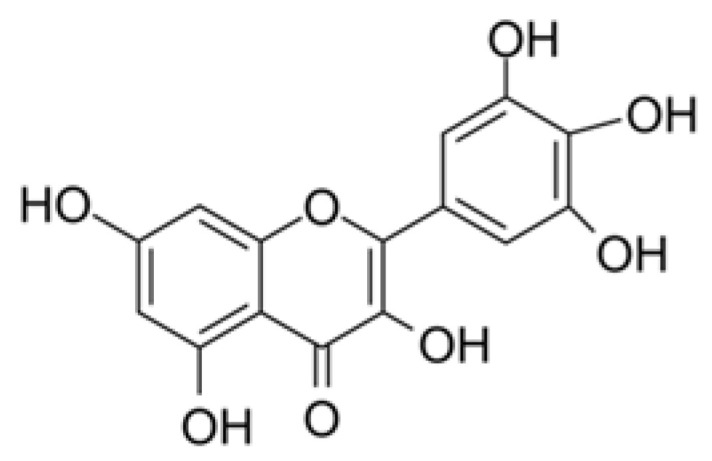
Myricetin chemical structure. Myricetin is a widespread plant-derived flavonoid with wide-ranging beneficial biological activities such as antioxidant, anticancer, and anti-inflammatory activities [83].

**Figure 14:**
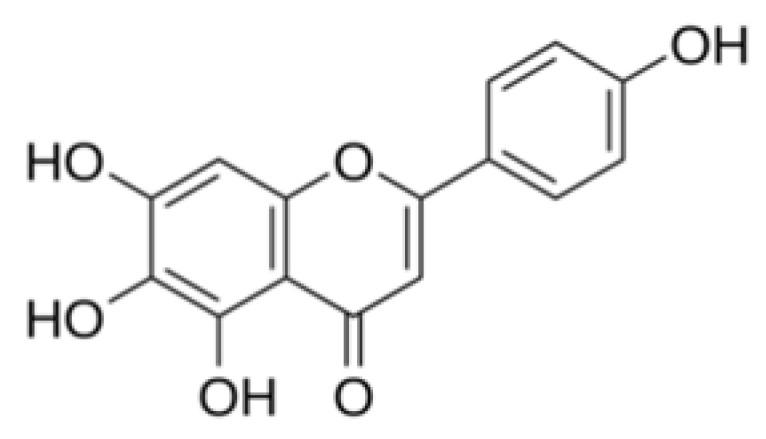
Scutellarein chemical structure [84].

**Figure 15:**
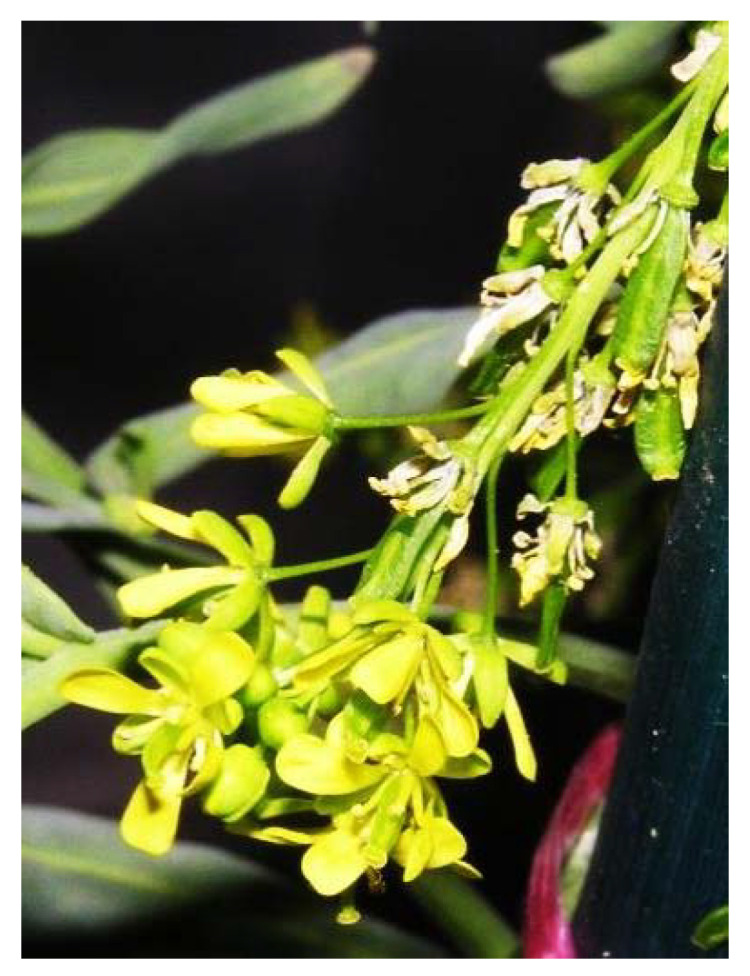
*Isatis indigotica* Fort. (Fam. Brassicaceae) [85].

**Figure 16:**
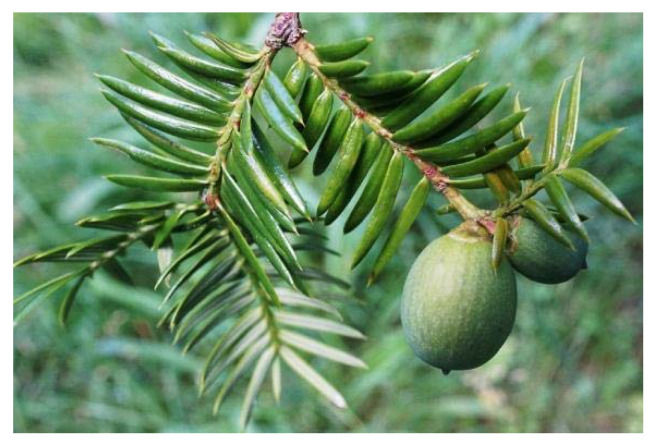
Japanese nutmeg-yew (*Torreya nucifera*) [86].

Natural antagonists of SARS-CoV enzymes, e.g., nsP13 helicase and 3CL protease, have been described, including myricetin (Figure 13) (a flavonoid polyphenolic molecule with antioxidant effects detected in fruits and vegetables), scutellarein (Figure 14) (a flavone occurring in the roots, stems, and flowers of *Scutellaria lateriflora*, a perennial member of the Lamiaceae, and other *Scutellaria* species, as well as *Asplenium belangeri*), and phenolic compounds from dyer’s woad (*Isatis indigotica*) (Figure 15) and Japanese nutmeg-yew (*Torreya nucifera*) (Figure 16) [26,51,55]. 

**Figure 17:**
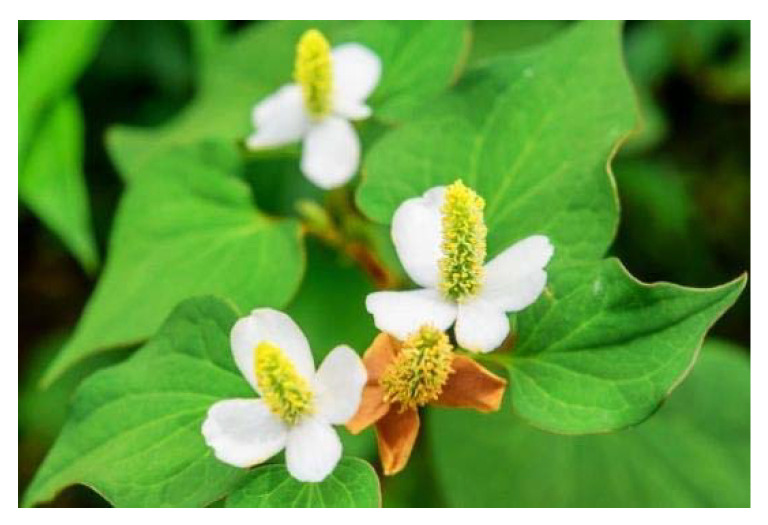
Houttuynia cordata [87].

Many natural anti-CoV phytomedicines include an aqueous extract of fish mint (*Houttuynia ordata*) (Figure 17), which has been demonstrated to mediate several antiviral mechanisms against SARS-CoV, e.g., inhibition of viral RNA-dependent RNA polymerase and suppression of the function of the viral 3CL protease [53]. 

Herbal preparations have been used as traditional medicines to ameliorate several illnesses. Some plant extracts were revealed to inhibit virus replication [88]. While medicinal plants, aromatic herbs, and volatile oils are known for their antibacterial and antifungal properties, there are currently insufficient scientific data to assess nontoxic and effective means to use them as antiviral treatments

The strongest options for efficacious antiviral chemotherapeutics are certain compounds that function on different viral biosynthetic pathways. In the viral replication cycle, they suppress different processes, and therefore little or no viral progeny is created. These medications may work at small doses, which do not damage the host cell. They will deter viruses from multiplying, eventually curing the contaminated cells. Regrettably, replicating viruses can develop resistance to these particular medications. Virucidal medications, on the other hand, interact with the membrane shell of enveloped viruses and solubilize viral structural glycoproteins [17,53,55]. The complex metabolism of natural bioactives is at the basis of numerous therapeutic agents and has contributed to the development of new antivirals. Compared with pesticides, herbal antiviral medicines have been understudied. However, some scientific trials have begun to evaluate their effectiveness more specifically. Medicinal plants and their isolated components have shown antiviral effects against certain coronaviruses [29], and the mechanism of action (Table 2) of these traditional supplements is mainly by viral replication suppression [17,19,23,26,48].

## 6. Phytomedicine and Clinical Trials for Coronavirus Infections

Chinese medicinal plants may provide additional solutions for COVID-19 prevention in high-risk communities, based on previous documents and demonstration of SARS protection in humans, but additional labor-intensive studies are required to validate the potential preventive impact of Chinese traditional medicine.

A Cochrane Review investigating the results of alternative therapies used during the SARS epidemic suggested a combination of herbal and conventional medicine did not lower the mortality rate but concluded that it may improve the quality of life, reduce chances of deep lung infiltration, and lower the dose of medications like corticosteroids [28]. A total of 640 persons with SARS participated in the investigation, which included 12 Chinese herbs. In combination with Western drugs, there was no statistical evidence of Chinese herbs reducing mortality over Western medicines alone. However, two plants demonstrated the ability to improve symptoms, five plants enhanced corticosteroid absorption through penetration of the lung, four herbs minimized corticosteroid dosages, three herbs enhanced the quality of life of patients with SARS, and one herb shortened the duration hospitalization.

China is currently conducting more than 80 preclinical studies on prospective COVID-19 therapies as well, including a few trials using traditional Chinese herbs [100]. There are about 15 experiments identified in China’s database, with more than 2000 estimated participants involved in studies on a number of traditional Chinese therapies. One of largest studies is testing shuanghuanglian, a Chinese herbal medication that includes substances from the dried fruit lianqiao (*Forsythiae fructus*), which has reportedly been used to treat infections for more than two millennia. The study involves 400 patients, including an experimental group receiving a normal treatment rather than a placebo therapy.

According to recent research [101], herbal medicines, like herbs and oils, may have a part to play in counteracting COVID-19. Research investigating the use of Indian medications as a therapy for manifestations of COVID-19 has been reported. The research presents the molecular morphology of the virus, potential modes of action inside the target cells, genomic similarity between COVID-19 and SARS, syndrome similarity between COVID-19, SARS, MERS, and typical flu, existing diagnosis, current clinical studies, and conventional Indian herbal medicines that may be produced as treatments directly aimed at COVID-19.

Luo et al. [102] have reviewed historical and clinical research on traditional Chinese medicines to avoid and alleviate infections in order to provide support to health agencies in China for the treatment of COVID-19, SARS, and H1N1 influenza. They traced back the use of traditional Chinese medicines to circumvent infectious epidemics and pandemics to ancient times.

**Figure 18:**
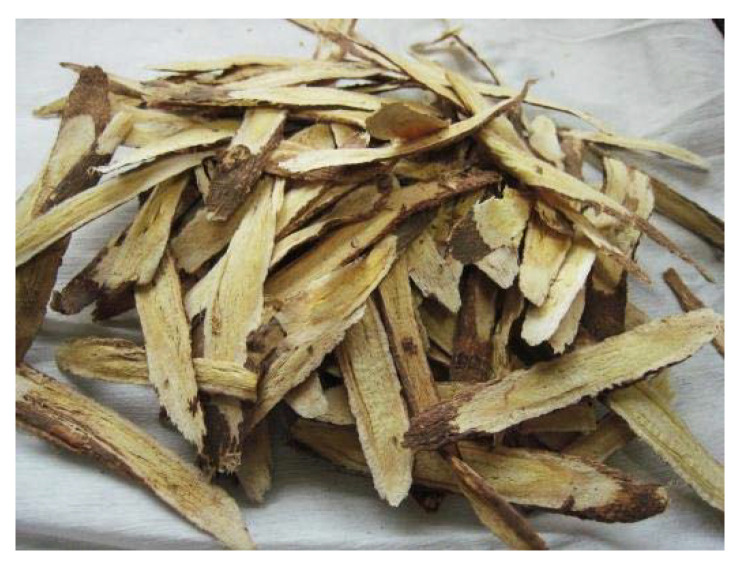
Astragali radix [103].

**Figure 19:**
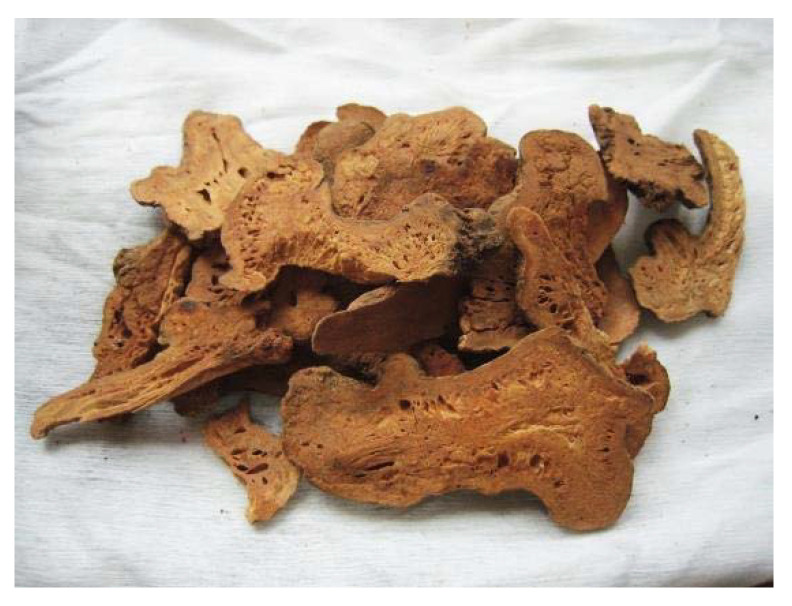
Rhizoma *Atractylodes macrocephalae* [104].

Based on these findings, three investigations followed focusing on Chinese medicine for the prevention of SARS. None one of the participants in these studies who received herbal remedies became infected with SARS. Based on those data, 23 territories in China released COVID-19 prevention strategies using appropriate herbal medicines used in Chinese medicine:

- *Radix astragali* (dried root of *Astragalus membranaceus* (Fisch.) (Figure 18) Bunge and *Astragalus mongholicus* Bunge (Fabaceae)) is a popular traditional Chinese medicine, and its active compounds may help fortify the immune system and decrease inflammation. *Astragalus* is occasionally also administrated as an injection in hospitals [102].
- *Radix glycyrrhizae* (dried roots and rhizomes of *Glycyrrhiza glabra*) or liquorice root is one of the 50 important plants used in phytomedicine [102].
- *Radix saposhnikoviae*, *Saposhnikovia divaricate,* recognized as fángfēng meaning “defend against the wind” in Chinese, is the single species in the genus *Saposhnikovia* [102].
- *Atractylodis macrocephalae* rhizome (Figure 19) is hailed as “the most essential Qi herb (vital energy in Chinese medicine) that tonifies and enhances the spleen”. It is the dried rhizome of *Atractylodes lancea* (Thunb.), *Atractylodes chinensis* Koidz, or any other nearby plant like *Japonica atractylodes* [32].
- *Lonicera japonica* Flos, member of the family Caprifoliaceae, is among the most widely used traditional medicines. It includes bioactive components such as caffeic acid derivatives, essential oils (EOs), flavonoids, iridoid glycosides, and terpenoids and it has anti-inflammatory, antimicrobial, anticancer, antioxidant, and immune-modulating properties [102].
- Golden Bell (*Fructus forsythia*) has long been recognized as a cure-all for patients who are especially vulnerable to skin infection. The plant has demonstrated broad-spectrum antibacterial activity and some suppression of influenza virus, leptospira, as well as other viruses. The plant also exhibits antipyretic and anti-inflammatory properties [32].

It has been reported that China has widely used traditional Chinese aromatic herbs and medicinal plants for the treatment of SARS successfully in several cases [102]. Nevertheless, there is no considerable confirmation yet on the clinical efficacy of these treatments in COVID-19 patients. In a study, 135 COVID-19 patients already received antiretroviral therapy in a previous clinical trial (135 received both immunotherapy and Kaletra^®^), while 59 received antibacterial therapy, and 36 were treated with anti-inflammatory agents (corticosteroids). In comparison, 124 patients were treated with Chinese traditional medicine [105].

The Chinese herbals used to treat COVID-19 mainly included glycyrrhiza (*G. glabra***)**, ephedra (*Ephedra sinica*), bitter almond (*Prunus dulcis* var. amara), gypsum, reed root (*Phragmites communis*), *Amomum*, and *Trichosanthes* (family Cucurbitaceae), and their principal function is to relieve cough and to improve immunity. This research recommended that patients should receive Kaletra^®^ (a combination of antiviral drugs lopinavir and ritonavir) very early and should also be treated by an association of Western and Chinese medicines, since Kaletra^®^ and traditional Chinese medicinal plants play a significant action in the management of viral pneumonia. Further scientific research is required to discover the mechanism of Kaletra^®^ and traditional Chinese medicinal plants in COVID-19 treatment.

## 7. Future Prospects

It is necessary to continue the development of efficacious antiviral chemotherapeutics that are cost-effective and with minimal side effects and which can also be used in combination with other drugs to improve the therapy of coronavirus-infected subjects. As protective vaccines and active antiviral drugs are not available for the treatment of several viruses, eliminating these viral infections seems hard and problematic. However, natural products serve as a tremendous source of biodiversity for developing innovative antivirals, with new structure–activity relationships, and potent medical and therapeutic approaches against viral infections.

A main problem surrounding antiviral drugs targeting specific viral proteins or genes is the capacity of a virus to rapidly mutate during replication, as observed for HIV and HSV [106], oseltamivir-resistant influenza viruses [107], and acyclovir- and nucleoside/nucleotide analog-resistant hepatitis B viruses [108]. There are several aspects that should be taken into account when assessing the antiviral activity of preparations of medicinal herbs, such as the extraction techniques used, since the highest level of antiviral activity is attained with acetone extracts or methanol fractions [109]. It is therefore appropriate, at the outset of a prospective study on aromatic herbal medicines, to identify the correct methodology for the preparation of the extracts, the parts of the plants to be used, the suitable season(s) for the collection of the materials, and the details of the application modality [110].

Although most research studies in this area are in their initial stages, additional research on the identification of active substances, the description of underlying mechanisms, as well as the analysis of efficiency and probable in vivo applications is recommended in order to assist the exploration of potent antiviral chemotherapeutics. Additional research should also investigate the possibility of combining these treatments with other natural ingredients or with standard medicines, as a multiple-target solution may help diminish the infection potential of drug-resistant virus strains. We trust that natural remedies, such as aromatic herbs, essential oils derived from medicinal plants, and pure oil compounds, will continue to play an important role and participate in the development and advancement of anti-coronavirus drugs.

## 8. Conclusions

Many viral infections are still lethal and/or are not yet treatable, even though some can be kept under control with life-prolonging agents, which, however, are expensive and outside the reach of most people. Thus, the discovery and development of safe, effective, and low-cost antiviral molecules is among the top universal urgencies of drug research.

Therefore, scientists and researchers from divergent medical fields are studying aromatic herbs and ethnomedicinal plants, with an eye to their applicability as antiviral drugs. Widespread research on ethnopharmacology and phytomedicine for the last 50 years resulted in the discovery of antivirals from natural products. Various traditional aromatic herbs and medicinal plants have been described as having strong and potent antiviral properties. Volatile oils, aqueous and organic extracts have, in general, demonstrated similar successful properties.

Considering the significant number of traditional medicinal plants that have provided good outcomes, it would seem reasonable to assume that these products contain different types of antiviral compounds. A characterization of secondary metabolites will reveal further health benefits. Therefore, the common usage of many traditional medicines for the prevention of viral infections is warranted. Eventually, the discovery and development of new antiviral agents from medicinal plants and herbs to control the threats presented by certain pathogenic viruses, such as the 2019-nCoV, is critical.

## Figures and Tables

**Table 1:** Studies describing the antiviral potential of different medicinal plants or isolated pure compounds against different strains of coronavirus (Cov). SARS, Severe Acute Respiratory Syndrome.

Coronavirus Strains	Plant Species or Isolated Compound	References
SARS-CoV	*Lycoris radiata*	Li et al. [23]
	*Artemisia annua* *Pyrrosia lingua* *Lindera aggregata* *Isatis indigotica*	Lin et al. [26]
	*Boenninghausenia sessilicarpa*	Yang et al. [43]
	*Lonicera japonica* *Eucalyptus* spp. *Panax ginseng*	Wu et al. [31]
Bovine coronavirus (BCV)	*Amelanchier alnifolia* *Cardamine angulata* *Rosa nutkana* *Verbascum Thapsus*	McCutcheon et al. [29]
SARS-CoV (Hong Kong strain)	*Dioscorea batatas* *Cassia tora* *Taxillus chinensis*	Wen et al. [44]
10 strains of SARS-CoV in fRhK4 cell line	Glycyrrhizin (*Glycyrrhiza uralensis)* Baicalin (*Scutellaria baicalensis)*	Chen et al. [15]
HCoV-229E	Mulberry (*Morus alba* var. *alba, Morus alba* var. *rosa*, and *Morus rubra*)	Thabti et al. [45]
	*Calophyllum blancoi*	Shen et al. [46]
	*Pelargonium sidoides*	Michaelis et al. [47]
	Saikosaponins (*Bupleurum* spp., *Heteromorpha* spp., *Scrophularia scorodonia)*	Cheng et al. [17]
SARS-CoV BJ01	*Galla chinensis*	Yi et al. [48]
SARS-CoV FFM1	Glycyrrhizin and glycyrrhetinic acid found in: *Glycyrrhiza radix*	Hoever et al. [18]
	*Laurus nobilis* Essential oil *fGentiana scabra*	Loizzo et al. [49]
SARS-CoV PUMC01 F5	*Cinnamomum* sp.	Zhuang et al. [50]
SARS-CoV helicase non-structural protein 13 (nsP13)	*Scutettaria baicalensis*	Yu et al. [51]
SARS-CoV 3CLpro	*Rheum palmatum*	Luo et al. [52]
	*Houttuynia cordata*	Lau et al. [53]
SARS-CoV CLpro	*Salvia miltiorrhiza*	Park et al. [54]
	*Torreya nucifera*	Ryu et al. [55]
SARS-CoV PLpro	*Broussonetia papyrifera*	Park et al. [56]
	*Psoralea corylifolia*	Kim et al. [57]
HCoV-NL63	*Strobilanthes cusia* leaf	Tsai et al. [30]
	*Sambucus formosana*	Weng et al. [58]
HCoV-OC43 HCoV-299E HCoV-NL63	Griffithsin (*Griffithsia* sp.)	O’Keefe et al. [59]

**Table 2:** List of medicinal plants or isolated active compounds inhibiting Coronaviruses.

Medicinal Plants (Phytochemicals or Compounds)	Common Name	Antiviral Mechanism	IC_50_ or EC_50_ Value	References
*Rosa nutkana*	Nootka Rose or wild Rose	Inhibition or reduction of the activity of enteric coronavirus—unidentified mechanisms.	-	McCutcheon et al. [29]
*Amelanchier alnifolia*	Saskatoon or pacific serviceberry or western serviceberry		-	
Luteolin		Blocking the viral entry of HIV-luc/SARS pseudo-type virus.	9.02 μM	Yi et al. [48]
*Lycoris radiata*	Red spider lily	Inhibition or reduction of viral attachment and penetration.	2.4 ± 0.2 μg/mL	Li et al. [23]
*Artemisia annua*	Sweet wormwood		34.5 ± 2.6 μg/mL	
*Pyrrosia lingua*	Tongue Fern		43.2 ± 14.1 μg/mL	
*Lindera aggregata*	Spicewood		88.2 ± 7.7 μg/mL	
*Isatis indigotica* (Beta-sitosterol)	Chinese Woad or dyer’s woad	Inhibition of nsP13 helicase and 3CL-like protease.	1.210 μM	Lin et al. [26]
Black tea (Theaflavin)		Inhibition of 3C-like protease of SARS-CoV.	9.5 μM	Chen et al. [89]
*Bupleurum marginatum*	Margined Chinese Thoroughwax	Interfering with early stages of viral replication, such as the penetration of the virus into the target cells. Some flavonoids are metabolized within the body into phenolate ions, inhibiting viral polymerase function, and connecting with viral nucleic acid or viral cuspid proteins. That tends to lead to viral replication being inhibited or reduced.	-	Cheng et al. [17]
*Astragalus membranaceus*	Mongolian milkvetch or Chinese *astragalus*	Immunomodulatory effects by increasing the number of lymphocytes and the proportion of CD4^+^ lymphocytes.	-	Yuan et al. [90]
Saikosaponins B2		Inhibition of viral attachment and penetration steps of HCoV-22E9.	1.7 ± 0.1 μM/L	Cheng et al. [17]
Curcumin		Inhibition of 3CL protease.	40 μM	Wen et al. [91]
*Rheum officinale*	Chinese rhubarb	Inhibition of the interaction between SARS-CoV S protein and angiotensin-converting enzyme 2 (ACE2).	1 to 10 μg/mL	Ho et al. [92]
*Polygonum multiflorum*	Tuber fleeceflower			
*Houttuynia cordata*	Fish mint or Chameleon-plant	Inhibition of 3CL-like protease and viral polymerase, and RNA-dependent RNA polymerase (RdRp) which are key enzymes involved with virus functions. Stimulate the proliferation of splenic lymphocytes which are necessary immune cells for fighting infection. Increase the proportion of CD4^+^ and CD8^+^ T cells necessary to fight viral infection.	-	Lau et al. [53] Kumar et al. [93]
*Torreya nucifera* (Amentoflavone)	Japanese nutmeg-yew or Japanese torreya	Inhibition of nsP13 helicase and 3CL protease.	8.3 μM	Ryu et al. [55]
*Verbascum Thapsus* (Verbascoside)	Great Mullein or *Common* mullein	Active ingredients decrease inflammation during respiratory infection.	-	Speranza et al. [94]
Herbal extracts (*Gentiana scabra*, *Dioscorea batatas*, *Cassia tora*, *Taxillus chinensis*, *Cibotium barometz*)		Inhibition of 3CL-like protease.	39 μg/mL and 44 μg/mL (two extracts of Cibotium barometz)	Wen et al. [44]
*Glycyrrhiza glabra* (Licorice Root)	Liquorice or Sweetwood	In vivo anti-inflammatory effect in the lungs by a glycoside known as LicoA.	-	Chu et al. [95]
*Ruscus aculeatus*	Butcher’s broom, knee holly or piaranthus	In vivo protection of lungs from inflammatory injury by the active ingredient (Ruscogenin, steroid sapogenin). Decreases of cerebral ischemia-induced blood–brain barrier dysfunction. Anti-inflammatory and anti-thrombotic properties.	-	Sun et al. [96]
Myricetin		3CL protease inhibition of SARS-CoV.	-	Yu et al. [51]
*Sambucus nigra*	Blue elder, common elder or Elderberry	Inhibition of chicken coronavirus strain if given at an early stage of infection.	-	Chen et al. [97]
*Psoralea corylifolia* (Bavachinin)	Babchi	Inhibitions of papain-like protease (PLpro).	38.4 ± 2.4 μM	Kim et al. [57]
*Hypericum perforatum*	Perforate St John’s wort or common Saint John’s wort	Inhibition of mRNA expression in Avian coronavirus infectious bronchitis virus (IBV).		Chen et al. [98]
*Sambucus formosana*	Blue elder, *common* elder or elderberry	Inhibition of chicken coronavirus strain and coronavirus NL63 by interfering with the viral envelopes, rendering them non-infectious.	-	Weng et al. [58]
Lycorine		Inhibition of cell division of different strains of coronaviruses (HCoV-OC43, HCoV-NL63, MERS-CoV, and MHV-A59).	0.15–0.31 μM.	Shen et al. [99]

## Abbreviations

ACE2: Angiotensin-Converting Enzyme 2; BCV: Bovine Coronavirus; CDC: US Centers for Disease Control and Prevention; CoV: Coronavirus; COVID-19: Coronavirus Disease 2019; EC_50_: half maximal effective concentration; EOs: Essential Oils; H1N1: Hemagglutinin Type 1 and Neuraminidase Type 1; HIV: Human Immunodeficiency Virus; HSV: Herpes Simplex Virus; IBV: Infectious Bronchitis Virus; IC_50_: Median Inhibitory Concentration; MERS-CoV: Middle East Respiratory Syndrome Coronavirus; nsP13: non-structural protein 13; RNA: RiboNucleic Acid; RSV: Respiratory Syncytial Virus; SARS-CoV: Severe Acute Respiratory Syndrome-Coronavirus; WHO: World Health Organization.
